# Adipose stem cell-derived extracellular vesicles ameliorates corticosterone-induced apoptosis in the cortical neurons via inhibition of ER stress

**DOI:** 10.1186/s13287-022-02785-4

**Published:** 2022-03-21

**Authors:** Sung-Ae Hyun, Young Ju Lee, Sumi Jang, Moon Yi Ko, Chang Youn Lee, Yong Woo Cho, Ye Eun Yun, Byoung-Seok Lee, Joung-Wook Seo, Kyoung-Sik Moon, Minhan Ka

**Affiliations:** 1 Department of Advanced Toxicology Research, Korea Institute of Toxicology, KRICT, Daejeon, 34114 Republic of Korea; 2 Department of Materials Science and Chemical Engineering, Hanyang University, Ansan, 15588 Republic of Korea

**Keywords:** Adipose stem cell-derived extracellular vesicles (A-EVs), Corticosterone, Apoptosis, ER stress, Cortical neurons

## Abstract

**Background:**

Corticosterone (CORT) can induce neuronal damage in various brain regions, including the cerebral cortex, the region implicated in depression. However, the underlying mechanisms of these CORT-induced effects remain poorly understood. Recently, many studies have suggested that adipose stem cell-derived extracellular vesicles (A-EVs) protect neurons in the brain.

**Methods:**

To investigated neuroprotection effects of A-EVs in the CORT-induced cortical neurons, we cultured cortical neurons from E15 mice for 7 days, and the cultured cortical neurons were pretreated with different numbers (5 × 10^5^–10^7^ per mL) of A-EVs (A-EVs^5^, A-EVs^6^, A-EVs^7^) for 30 min followed by administration of 200 μM CORT for 24 h.

**Results:**

Here, we show that A-EVs exert antiapoptotic effects by inhibiting endoplasmic reticulum (ER) stress in CORT-induced cortical neurons. We found that A-EVs prevented neuronal cell death induced by CORT in cultured cortical neurons. More importantly, we found that CORT exposure in cortical neurons resulted in increased levels of apoptosis-related proteins such as cleaved caspase-3. However, pretreatment with A-EVs rescued the levels of caspase-3. Intriguingly, CORT-induced apoptosis involved upstream activation of ER stress proteins such as GRP78, CHOP and ATF4. However, pretreatment with A-EVs inhibited ER stress-related protein expression.

**Conclusion:**

Our findings reveal that A-EVs exert antiapoptotic effects via inhibition of ER stress in CORT-induced cell death.

## Background

Extracellular vesicles (EVs) are lipid bilayer membrane particles endogenously released from many different cell types under both normal and pathological conditions [1]. Endogenously released EVs carry various cargoes, including DNAs (mitochondrial DNA, single-stranded DNA, double-stranded DNA), RNA species (mRNA, microRNA, long noncoding RNA, and other RNA species), and membrane proteins, including receptors and major histocompatibility complex (MHC) molecules, which mediate intercellular communication through the transport and exchange of these cargoes [2, 3]. Due to this biological activity, EVs have innate therapeutic potential in tumorigenesis, the spread of viruses, neurodegenerative diseases and infectious diseases [4]. Moreover, the therapeutic role of EVs has been shown in neuroinflammation, neurodegeneration, cancers and disorders that affect the central nervous system (CNS) [5, 6]. These effects are because EVs play an important role in the nervous system, not only providing communication between neurons and glial cells in the brain but also causing interconnection of body systems and the CNS [7]. Therefore, various studies are conducted to assess the therapeutic role of EVs in these CNS-related diseases [8–11].

The physiological stress response involves the rapid activation of the sympatho-adrenal axis and the release of catecholamines from the adrenal medulla and induces the release of glucocorticoids [12]. Exposure to these persistent psychological stresses leads to hyperactivity of the hypothalamic–pituitary–adrenal (HPA) axis and elevated glucocorticoid levels [13, 14]. Glucocorticoids are a class of steroid hormones produced from the adrenal cortex in the form of corticosterone (CORT) in rodents and cortisol in humans [15] and are critical for the regulation of development, metabolism and immune functions [16]. Specifically, prolonged exposure to CORT leads to neuronal damage, particularly in the hippocampus, which is enriched with corticosteroid receptors [14, 17, 18]. However, the definite cellular mechanisms underlying CORT-induced neuronal cell damage have not been fully elucidated. Previous studies have proven that persistent exposure of nerve cells to high concentrations of CORT causes DNA damage, induces differential protein activation and consequently leads to nerve cell apoptosis [19, 20]. Accumulating reports have shown that oxidative stress may contribute to neuronal injury induced by CORT [21–23]. Moreover, this oxidative imbalance was reported to trigger endoplasmic reticulum (ER) dysfunction [24, 25].

In the present study, we aimed to determine whether CORT is responsible for apoptosis in primary cultured cortical neurons and to investigate the protective effects of A-EVs. In addition, we discuss whether the neuroprotective effects of EVs occur via inhibition of ER stress-mediated apoptotic pathways.

## Methods

### Reagents

The CORT (CAS number: 50-22-6, catalog number: 27840, Sigma-Aldrich, St. Louis, MO) was more than 99% pure and dissolved in dimethyl sulfoxide (DMSO) (CAS number: 67-68-5, catalog number: D8418, Sigma-Aldrich, St. Louis, MO).

### Primary neuronal cultures

Primary neuronal culture was described previously [26, 27]. In brief, cerebral cortex from E15 mice was isolated under dissecting microscope and were treated with 0.05% trypsin-EDTA (25300054, Gibco) for 10 min at 37 °C. The enzyme reaction was neutralized by sequential washes with neat FBS and culture medium, neurobasal (21103-049, Gibco) containing B27 (A35828-01, Gibco) and N-2 Supplements (17502-048, Gibco), 2 mM L-Glutamine (25030-081, Gibco), and Penicillin–Streptomycin (100 U/ml and 100 μg/ml, respectively; 15140-122, Gibco). After dissociation by gentle pipetting, neurons were counted and plated (1 × 10^5^ cells/cm^2^) onto coated (50 μg/ml poly-D-lysine and 10 μg/ml laminin) coverslips or culture plate. Following 7 days of in vitro culture, the cortical neurons were pretreated with A-EVs (for 30 min) or ISRIB (1 μM, for 1 h). After that, CORT (200 μM, unless otherwise stated) were treated for 24 h.

### Preparation and characterization of A-EVs

#### Cell culture and A-EVs isolation from conditioned medium

Primary human ADSCs were purchased from CEFO Bio Co., Ltd (Seoul, Korea) and maintained in growth medium (Minimum Essential Medium (MEM)-α containing 10% fetal bovine serum (FBS), 20 μg/mL bFGF and 10 μg/mL Gentamicin) at 37 °C in 5% CO_2_. After reaching 80–90% confluence, the medium was changed to conditioned medium (phenol red free Dulbecco’s Modified Eagle Medium (DMEM) containing 1% sodium pyruvate, 1% L-glutamine and 10 μg/mL Gentamicin) for 24 h. 500 mL of collected conditioned media (CM) was pre-filtered using a 0.2-μm bottle top filter to remove cell debris and large impurities. The filtered CM was purified and concentrated by using tangential flow filtration (TFF) systems (Repligene) with a hollow filter unit (300-kDa MWCO). While the media circulated in the TFF systems, small molecules less than 300 kDa are filtered out, and A-EVs were concentrated. To obtain a high-purity exosome solution, the concentrated solution was diluted by phosphate-buffered saline (PBS) and re-circulated in the TFF systems. Eventually, small molecules were washed out, and 10–15 mL of concentrated A-EVs were obtained. Isolated A-EVs were aliquoted and stored at below − 70  °C until use.

#### Nanoparticle tracking analysis (NTA)

The particle concentration and size distribution of A-EVs were measured by nanoparticle tracking analysis (Nanosight LM10, malvern Instruments Ltd). A-EVs were resuspended in PBS to obtain a concentration within the recommended measurement range (20–30 particles/frame), corresponding to dilutions from 1:10 to 1:100 depending on the initial sample concentration. The software settings for analysis were as follows: detection threshold 3; temperature between 22 °C; number of frames 30 and measurement time 30 s. The size distribution and particle concentration each represent the mean of three individual measurements.

#### Transmission electron microscopy

To visualize the morphology of A-EVs, transmission electron microscopy image analysis was performed. A-EVs were fixed with 0.5% glutaraldehyde solution overnight. The fixed A-EVs were centrifuged at 13,000×*g* for 3 min. Then the supernatant was removed. Next, the pellets were dehydrated in absolute ethanol for 10 min and placed on formvar–carbon-coated copper grids (TED PELLA, Inc., Redding, CA, USA). The grids were stained with 1% phosphotungstic acid for 1 min and then washed several times with absolute ethanol solution. The grids were thoroughly dried off and then analyzed with a JEM-2100 F field emission electron microscope (JEOL Ltd., Japan).

#### Flow cytometry analysis

Flow cytometry analysis (FACS) of A-EVs was performed using a commercially available Exo-Flow capture kit (System Biosciences, CA, USA) according to the manufacturer’s protocol. Briefly, isolated A-EVs were captured on microbead with CD9, CD63, CD81, GM130 and Calnexin antibodies provided in the kit. The A-EVs-microbead complexes were stained by Exo-FITC and analyzed by FACS (Novocyte Flow Cytometer, ACEA Bioscience, Inc., MA, US). Data acquisition and analysis were performed using NovoExpress software.

### Cell viability assay (WST-8 assay)

Cell viability assay was described previously [28] in brief, for analysis of cell viability, 1 × 10^4^ cells/well were seed in a 96 well plates and incubated for 24 h at 37 °C under humidified conditions (5% CO_2_ atmosphere). Then, cells were treated with CORT at concentrations of 50, 100, 200, 250 and 500 μM for 24 h. Then, EZ-Cytox Kit (WST-8 assay; DoGen, Seoul, Korea) was added to each well at a final concentration of 0.5 mg/mL, and the cells were incubated for 2 h at 37 °C under humidified conditions (5% CO_2_ atmosphere). Finally, absorbance was measured at 450 nm using a microplate reader (GloMax, Promega, WI, USA).

### Immunoblotting

Western blotting was performed as described previously [29, 30]. Tissue lysates from hippocampal region were prepared using RIPA buffer and the sample was centrifuged at 12,000 rpm for 10 min at 4 °C, then the supernatant was collected and protein content was determined by Pierce BCA Protein Assay Kit (Thermo Fisher Scientific, Waltham, MA, USA) following the manufacturer's protocol. Proteins were separated on 8%, 10% or 15% SDS-PAGE gradient gel and transferred onto PVDF transfer membrane (Thermo Fisher Scientific, Waltham, MA, USA). Then the membrane was incubated with rabbit anti-Caspase-3 (#9662, Cell Signaling Technology, Danvers, MA, USA), mouse anti-BAX (SC-20067, Santa Cruz Biotechnology, Dallas, TX, USA), mouse anti-Bcl2 (SC-7382, Santa Cruz Biotechnology, Dallas, TX, USA), rabbit anti-GRP78 (ab21685, Abcam, Cambridge, UK), rabbit anti-CHOP (MBS9606693, MyBioSource, San Diego, CA, USA), mouse anti-ATF4 (SC-390063, Santa Cruz Biotechnology, Dallas, TX, USA) and mouse anti-β-actin (A5316, Thermo Fisher Scientific, Waltham, MA, USA) at 4 °C overnight. Appropriate secondary antibodies conjugated to HRP were used (Thermo Fisher Scientific, Waltham, MA, USA) and the ECL reagents (Thermo Fisher Scientific, Waltham, MA, USA) were used for immunodetection. For quantification of band intensity, blots from 3 independent experiments for each molecule of interest were used. Signals were measured using ImageJ software and represented by relative intensity versus control. β-actin was used as an internal control to normalize band intensity.

### Reverse transcription PCR

Reverse transcription PCR was performed as described previously [29]. RNA was extracted from cultured neurons using TRIZOL reagent (Thermo Fisher Scientific), and cDNA was synthesized from 1 µg of total RNA using oligo-dT and random hexamers using the Verso cDNA synthesis kit (Thermo Fisher Scientific). A measure of 1 µl of cDNA was used in reverse transcription PCR using Master Mix (Promega Life Sciences). The sequences of the primers used were GRP78 forward 5′-ACTTGGGGACCACCTATTCCT-3′ and reverse 5′-ATCGCCAATCAGACGCTCC-3′, ATF4 forward 5′-ATGGCGCTCTTCACGAAATC-3′ and reverse 5′-ACTGGTCGAAGGGGTCATCAA-3′, CHOP forward 5′-CTGGAAGCCTGGTATGAGGAT-3′ and reverse 5′-CAGGGTCAAGAGTAGTGAAGGT-3′, and GAPDH forward 5′-AGGTCGGTGTGAACGGATTTG-3′ and reverse 5′-TGTAGACCATGTAGTTGAGGTCA-3′.

### TUNEL assay and microscopy

TUNEL assay was performed according to manufacturer’s instructions (DeadEnd™ Fluorometric TUNEL System, catalog number: G3250, Promega, Madison, WI, USA) to detect cell death in the cultured cortical neuron. The assay stained in the green channel at 488 nm. DAPI was applied as a nuclear counterstain in the blue channel at 461 nm. Images were taken with an Olympus FV3000 fluorescent microscope and Olympus software. Exposure settings were adjusted to minimize oversaturation. For analyzing cultured cells, more than 20 fields scanned horizontally and vertically were examined in each condition. Cell numbers were described in figure legends. The calculated values were averaged, and some results were recalculated as relative changes versus control.

### Statistical analysis

Normal distribution was tested using the Kolmogorov–Smirnov test, and variance was compared. Unless otherwise stated, statistical significance was determined by one-way or two-way analysis of variance (ANOVA) followed by the Bonferroni post hoc test for multiple comparisons. Data were analyzed using GraphPad Prism (GraphPad Software, Inc. La Jolla, CA, USA) and presented as mean (±) SEM. P values were indicated in figure legends.

## Results

### CORT exposure induces neuronal apoptosis in cultured cortical neurons

**Fig. 1:**
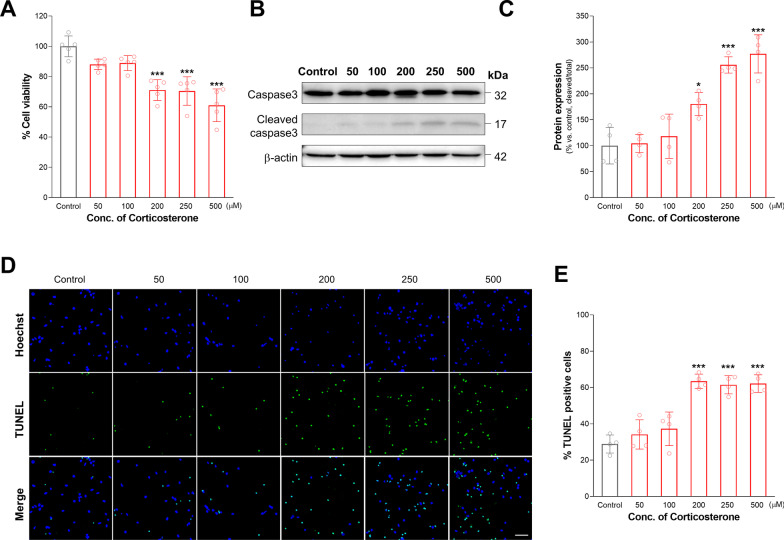
Corticosterone-induced apoptosis in cortical neurons. **A** Cell viability was measured after treatment with 50–500 μM corticosterone for 24 h in primary cultured cortical neurons. *n* = 6. **B** Expression of total or cleaved Caspase-3 expression was detected by immunoblot in corticosterone-treated cells. **C** Quantitative analysis was showed on cleaved Caspase 3 per total caspase 3 protein expression. *n* = 4. **D** Fluorescence imaging of TUNEL staining after corticosterone treated-cortical neurons. Scale bar = 50 μm **E** Quantitative analysis was performed for TUNEL positive cells. *n* = 4 Statistical significance was determined by ANOVA with Bonferroni correction test. Data are shown as relative changes versus controls. *p < 0.05; **p < 0.01 and ***p < 0.001

To examine the cytotoxic effects of CORT in cultured cortical neurons, we cultured cortical neurons from embryonic day 15 (E15) mice for 7 days, exposed the cortical neurons to varying doses of CORT (50, 100, 200, 250, 500 μM) for 24 h and assessed cell viability by WST-8 assays. As shown in Fig. 1A, lower concentrations (50–100 μM) of CORT for 24 h did not significantly change the cell viability, while higher doses (200–500 μM) decreased cell survival. Next, we assessed the level of cleaved caspase-3 in the lysates of the CORT-induced cortical neurons. We found that the levels of cleaved caspase-3 were dose-dependently increased by 81%, 156% and 177% after exposure to high doses (200–500 μM) in the CORT-induced cortical neurons (Fig. 1B, C). Finally, to confirm the cytotoxic effects of CORT in cultured cortical neurons, we performed TUNEL staining assays. We found that lower concentrations (50–100 μM) of CORT for 24 h did not significantly change the number of TUNEL-labeled cortical neurons. However, the number of TUNEL-labeled cortical neurons was dose-dependently increased by 120%, 113% and 116% after exposure to high doses (200–500 μM) of CORT (Fig. 1D, E). These results indicate that CORT exposure induced cytotoxicity in cultured cortical neurons.

### CORT exposure induces ER stress in cultured cortical neurons

**Fig. 2:**
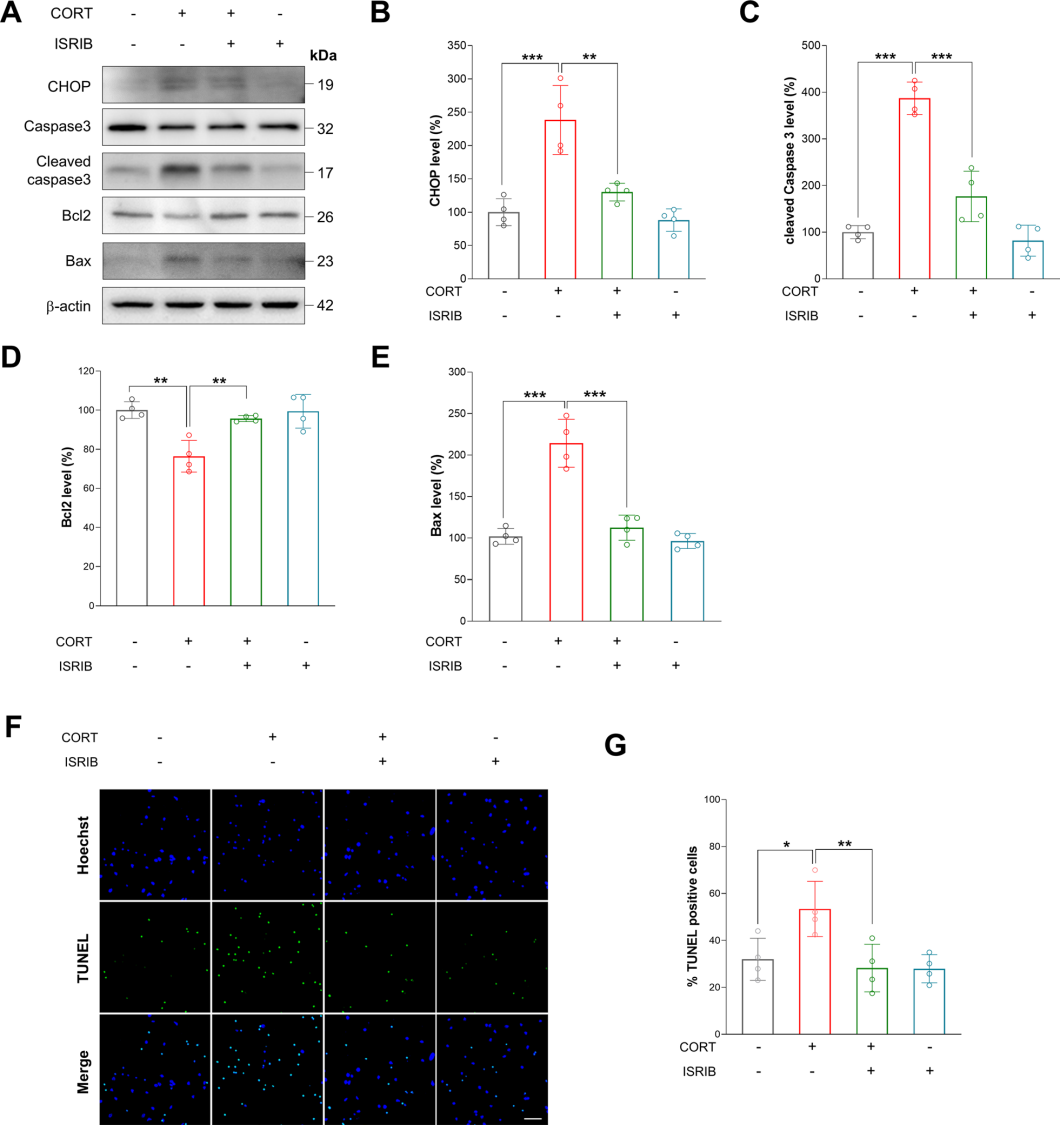
Corticosterone-induced apoptosis of cortical neurons is caused by ER stress. **A** ER stress or apoptosis-related proteins was measured by immunoblot in corticosterone or ISRIB-treated cells. **B** Quantification of CHOP protein levels shown in **A**. The relative expression of protein was normalized to β-actin. *n* = 4 **C** Quantification of cleaved caspase3/total caspase-3 protein levels shown in **A**. *n* = 4 **D** Quantification of Bcl2 protein levels shown in **A**. The relative expression of protein was normalized to β-actin. *n* = 4 **E** Quantification of Bax protein levels shown in **A**. The relative expression of protein was normalized to β-actin. *n* = 4 Statistical significance was determined by ANOVA with Bonferroni correction test. Data are shown as relative changes versus controls. *p < 0.05; **p < 0.01 and ***p < 0.001

To investigate whether ER stress is involved in CORT-induced apoptosis of cortical neurons, we cultured cortical neurons from E15 mice for 7 days and pretreated them with an ER stress inhibitor, ISRIB, for 1 h, followed by CORT exposure for 24 h. Then, we assessed the levels of the ER stress marker CHOP in the lysates of the cultured cortical neurons. We found that the CHOP level was increased by 138% in the CORT-induced cortical neurons (Fig. 2A, B). However, pretreatment with ISRIB restored CHOP expression in the CORT-induced cortical neurons (Fig. 2A, B). Next, we assessed the levels of apoptotic markers such as cleaved caspase-3, Bax and Bcl2 in the lysates of the cultured cortical neurons. The levels of cleaved caspase-3 and Bax were increased by 287% and 122%, respectively, in the CORT-induced cortical neurons (Fig. 2A, C, E). Moreover, we found that the level of Bcl2 was decreased by 26% in the CORT-induced cortical neurons (Fig. 2A, D). However, pretreatment with ISRIB rescued cleaved caspase-3, Bax and Bcl2 expression in the CORT-induced cortical neurons (Fig. 2A, C–E). Finally, to confirm ER stress-mediated apoptosis in the CORT-induced cortical neurons, we performed TUNEL staining assays. Pretreatment of cortical neurons with ISRIB significantly inhibited CORT-mediated apoptosis (Fig. 2F, G). These results show that CORT exposure activates neuronal apoptosis by inducing ER stress in cultured cortical neurons.

### A-EVs suppresses neuronal apoptosis in CORT-induced cortical neurons

**Fig. 3:**
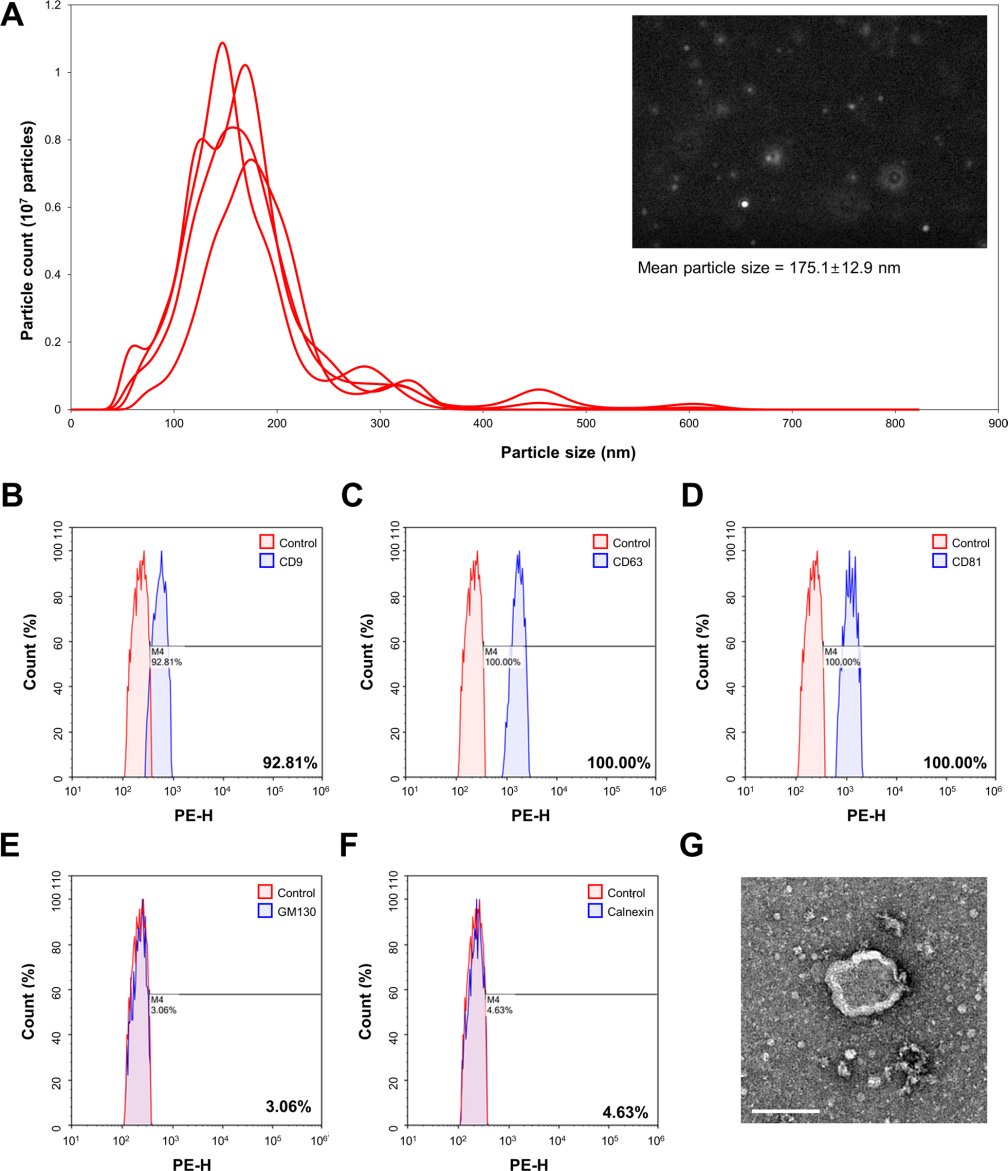
The characterization of A-EVs. **A** Particle size distribution of A-EVs measured by NTA. **B**–**F** Flow cytometry analysis of EV surface markers (CD9, CD63, CD81) and internal protein markers (GM130 and Calnexin). **G** TEM images of A-EVs. White bar represents 100 μm

**Fig. 4:**
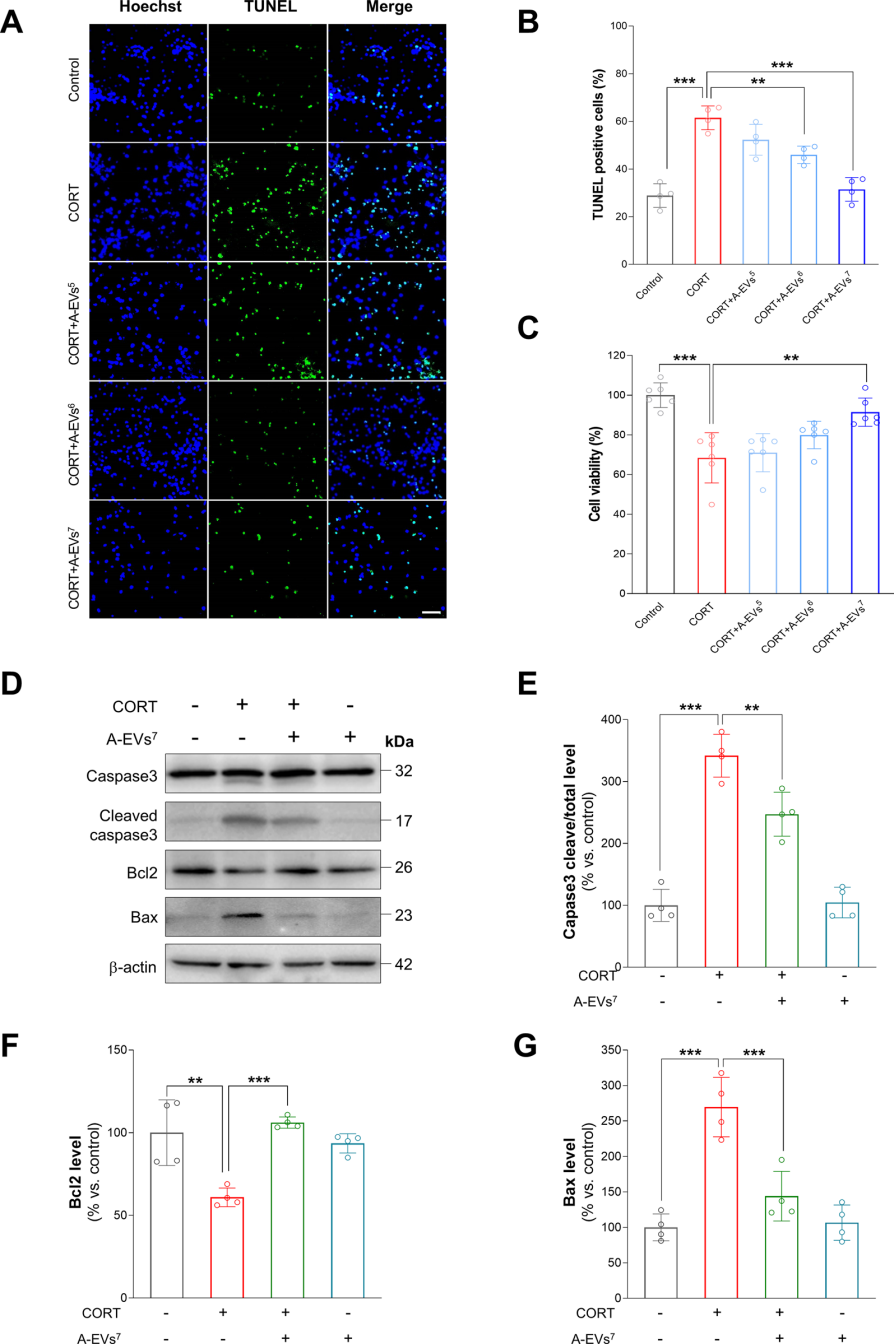
The protective effect of A-EVs against corticosterone-induced apoptosis in cortical neurons. **A** Representative fluorescence images of TUNEL staining in corticosterone with or without A-EVs-treated primary cultured cortical neurons. Scale bar = 50 μm **B** TUNEL-positive cells/nuclei by image were quantified. *n* = 4 **C** Cell viability was measured after treatment in corticosterone with or without A-EVs-treated for 24 h. *n* = 6 **D** Apoptosis-related proteins was measured by immunoblot in corticosterone or A-EVs-treated cells. **E** Expression of cleaved caspase3/total caspase-3 protein level was quantified. *n* = 4 **F** Quantification of Bcl2 protein levels. *n* = 4 **G** Quantification of Bax protein levels. *n* = 4. The expression of protein was normalized to β-actin. Statistical significance was determined by ANOVA with Bonferroni correction test. Data are shown as relative changes versus controls. *p < 0.05; **p < 0.01 and ***p < 0.001

Recent evidence has shown that A-EVs induce neuronal protection and enhance neurological recovery. Thus, we investigated whether A-EVs could induce neuronal protection in the CORT-induced cortical neurons. As shown in Fig. 3, the round spherical shape of the A-EVs was observed by TEM analysis, and their mean diameter was determined to be 175.1 nm. FACS analysis revealed that A-EVs were positive for EV markers, including CD9 (92.81%), CD63 (100.00%) and CD81 (100.00%), whereas negative expression of the non-EV markers GM130 (3.06%) and Calnexin (4.63%) were observed. Based on the concentration-dependent effects of CORT on neuronal toxicity in cultured cortical neurons, we cultured cortical neurons from E15 mice for 7 days, and the cultured cortical neurons were pretreated with different numbers (5 × 10^5^–10^7^ per mL) of A-EVs (A-EVs^5^, A-EVs^6^, A-EVs^7^) for 30 min followed by administration of 200 μM CORT for 24 h. Then, we assessed the number of apoptotic cells in the cultured cortical neurons by TUNEL staining. As expected, CORT exposure increased the number of apoptotic neurons by 113% compared with that of the controls (Fig. 4A, B). Importantly, pretreatment of neurons with A-EVs^6^ and A-EVs^7^ suppressed CORT-induced cell death in the cultured cortical neurons (Fig. 4A, B). Next, we assessed the cell viability by WST-8 assays. Consistently, CORT exposure decreased the cell viability by 32% compared with that of the controls (Fig. 4C). However, pretreatment of neurons with A-EVs^7^ rescued CORT-induced neuronal cell death (Fig. 4C). Finally, we assessed the levels of cleaved caspase-3, Bax and Bcl2 in the lysates of cortical neurons. We found that the levels of caspase-3 and Bax were increased by 242% and 170%, respectively, in the CORT-induced cortical neurons (Fig. 4D–F). However, pretreatment with A-EVs^7^ rescued the levels of cleaved caspase-3 and Bax (Fig. 4C–F). As expected, CORT exposure decreased the level of Bcl2 by 39% compared with the control (Fig. 4D, G). However, pretreatment of neurons with A-EVs^7^ restored the level of Bcl2 in the CORT-induced cortical neurons (Fig. 4D, G). These results suggest that A-EVs prevent neuronal cell death induced by CORT in cultured cortical neurons.

### A-EVs suppresses neuronal apoptosis by inhibition of ER stress in CORT-induced cortical neurons

**Fig. 5:**
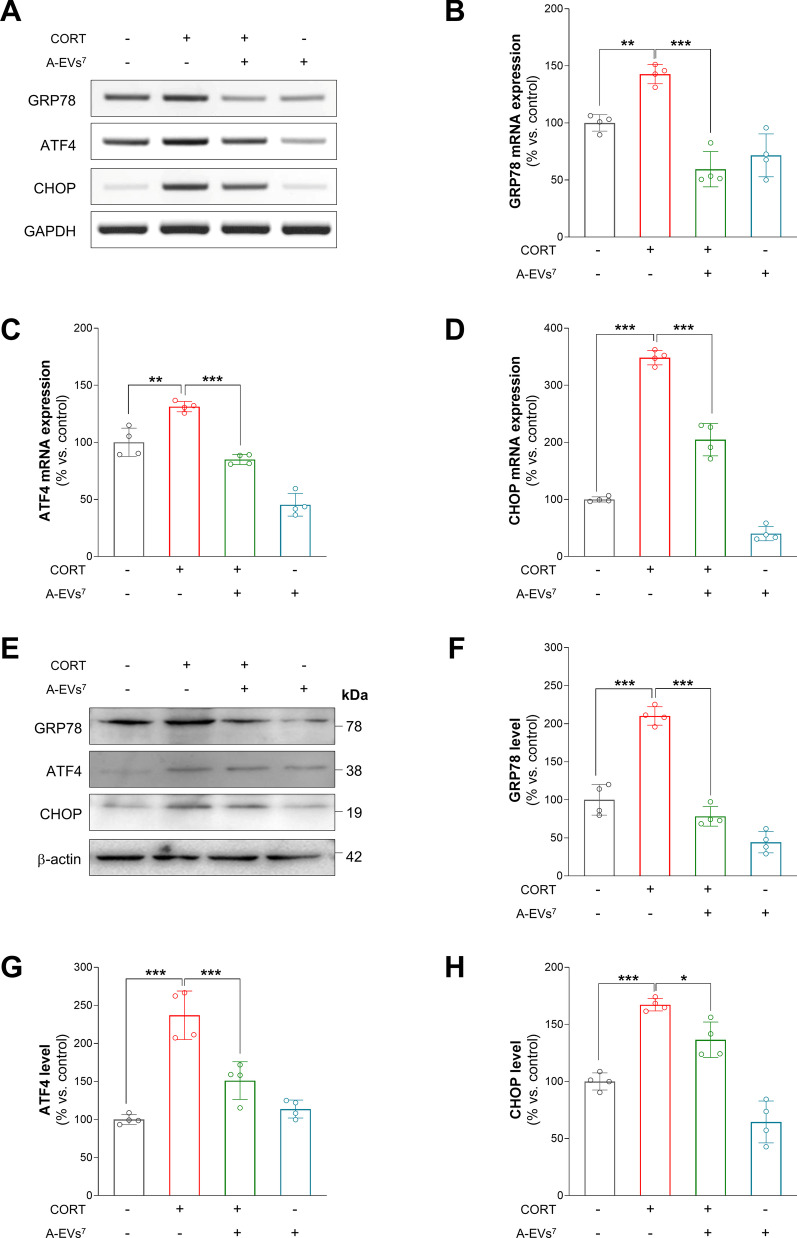
Effect of A-EVs on corticosterone-induced ER stress in cortical neurons. **A** A-EVs restores CORT-induced ER-stress related GPR78, ATF4, and CHOP mRNA levels. ER stress-related mRNAs, GRP78, ATF4, and CHOP, were measured by RT-PCR. **B** Quantification of GRP78 mRNA level shown. The fold change of GRP78 was normalized to GAPDH. *n* = 4. **C** Quantification of ATF4 mRNA level shown. The fold change of ATF4 was normalized to GAPDH. *n* = 4. **D** Quantification of CHOP mRNA was analyzed. The fold change of CHOP was normalized to GAPDH. *n* = 4 **E** EV ameliorated CORT-induced ER stress in cortical neurons. ER stress-related proteins, GPR78, ATF4 and CHOP, were measured by immunoblot. **F** Expression of GRP78 was quantified and normalized to β-actin. *n* = 4 **G** Expression of ATF4 level is analyzed. The expression of ATF4 was normalized to β-actin. *n* = 4 **H** Quantification of CHOP protein level was analyzed. The expression of protein was normalized to β-actin. *n* = 4 Statistical significance was determined by ANOVA with Bonferroni correction test. Data are shown as relative changes versus controls. *p < 0.05; **p < 0.01 and ***p < 0.001

Based on the antiapoptotic effects of A-EVs in CORT-induced cortical neurons, we investigated whether pretreatment with A-EVs^7^ could also lead to alterations in ER stress in CORT-induced cortical neurons. We cultured cortical neurons from E15 mice for 7 days, and the cultured cortical neurons were pretreated with A-EVs^7^ for 30 min followed by administration of 200 μM CORT for 24 h. Using RT-PCR, we first measured the transcript levels of ER stress-related proteins such as GRP78, ATF4 and CHOP in the cultured cortical neurons. We found that the GRP78, ATF4 and CHOP mRNA levels were increased by 42%, 30% and 245%, respectively, in the CORT-induced cortical neurons compared with the control neurons (Fig. 5A–D). However, pretreatment with A-EVs^7^ restored the GRP78, ATF4 and CHOP mRNA levels (Fig. 5A–D).

Next, we assessed the expression levels of GRP78, ATF4 and CHOP by immunoblotting of the cultured cortical neurons (Fig. 5E–H). Similarly, we found that the protein levels of GRP78, ATF4 and CHOP increased by 108%, 124% and 68%, respectively, in the CORT-induced cortical neurons compared with the control neurons (Fig. 5E–H). Importantly, pretreatment with A-EVs^7^ rescued GRP78, ATF4 and CHOP protein expression in the CORT-induced cultured cortical neurons. These results suggest that A-EVs inhibit ER stress in cultured cortical neurons.

## Discussion

**Fig. 6:**
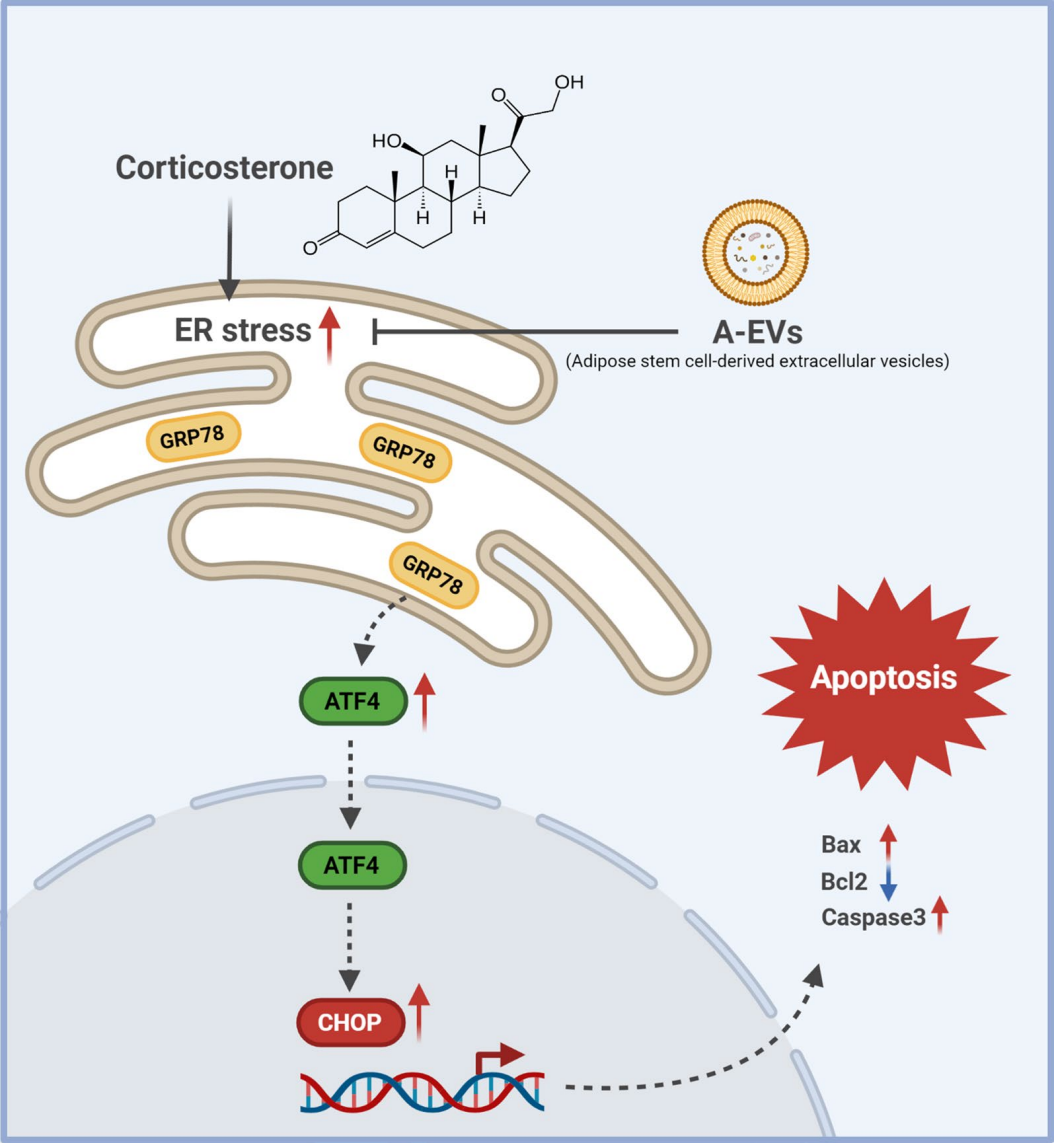
A schematic model illustrating an effect of A-EVs on CORT-induced apoptosis in the cortical neurons via inhibition of ER stress

In this study, we show that CORT induces neuronal apoptosis by activating ER stress and that pretreatment with A-EVs ameliorates CORT-induced apoptosis. In cortical neurons, the activation of ER stress plays an essential role in CORT-induced neuronal apoptosis. However, interestingly, A-EVs suppressed CORT-induced neuronal apoptosis by inhibiting ER stress (Fig. 6). Our results provide novel insights into molecular targets for CORT-induced neuronal cell death. Moreover, elucidation of the mechanisms of CORT-induced neuronal cell death could have implications for the future development of antiapoptotic drugs.

The corticosteroid-type hormone CORT is produced in the adrenal cortex. The CORT circulates the whole body via bloodstream, and its persistent exposure exerts a toxic effect on neurons [31, 32] and induces depression- and anxiety-like behaviors in rodents [14, 33]. More specifically, CORT causes synaptic abnormalities by altering the dendritic architecture of cultured cortical and hippocampal neurons [14, 18, 34, 35], and it also suppresses adult neurogenesis in the dentate gyrus [36, 37] and embryonic neural stem cells proliferation [38]. Furthermore, CORT induces apoptotic neuronal death [15]. Here, CORT exposure induced a significant increase of TUNEL positive cells and pro-apoptotic proteins in mouse cortical neurons. ER stress is one of the triggers for apoptotic cell death [39, 40], which is also observed in CORT-exposed PC12 cells [41] and hippocampal neurons [42]. In this study, the involvement of ER stress in CORT-induced apoptosis was revealed via measuring the signaling pathway proteins expression and inhibitor-mediated restoration. Upon ER stress, accumulation of unfolded proteins leads to dissociation of GRP78, a key chaperone in ER, from ER transmembrane receptors. PERK is one of these receptors, and after dissociation, it is activated by auto-phosphorylation [43]. Subsequently, activated PERK phosphorylates eIF2α, a key factor of the integrated stress response [44]. Phosphorylated eIF2α blocks translation except for some specific targets, including stress-induced transcription factor ATF4. During mild stress, ATF4 promotes the expression of pro-survival genes, including GRP78 [45] to restore the stress condition. However, if the stress is prolonged, ATF4 induces CHOP expression, which is a crucial pro-apoptotic factor and results in apoptotic cell death. [46]. CORT exposed neurons exhibited elevation of GRP78, ATF4, and CHOP expression. ISRIB is an inhibitor of phosphorylated eIF2α actions [47]. ISRIB pretreatment reverted CORT-induced apoptosis in TUNEL assay and apoptotic protein levels. These results suggest that the CORT induces the cell death in cortical neurons via ER stress-mediated apoptosis.

In various tissues and cells, mesenchymal stem cell (MSC)-derived EVs alleviate ER stress and consequently prevent apoptosis. Placenta-derived MSC-EVs protected ischemic-reperfusion injured kidneys through the suppression of ER stress [48]. Bone marrow MSC-EVs attenuated ER stress-mediated apoptosis by activating the AKT and ERK signaling in intervertebral disc cells [49]. Umbilical cord MSC-EVs protect the pancreatic beta-cell from hypoxia-induced ER stress and apoptosis via miR-21which by inhibiting p38 MAPK phosphorylation [50]. Among the MSCs, abundance and accessibility are advantages of adipose-derived MSCs [51]. However, ER stress-related studies of its EVs were still limited. Here, A-EVs pretreatment attenuated CORT-mediated apoptosis, similar to ISRIB pretreatment. Expression of GRP78, ATF4 and CHOP also reduced by A-EVs pretreatment. Our findings indicate that A-EVs protects cortical neurons from CORT-induced apoptosis via suppressing the ER stress, and these further suggest the potential of A-EVs in the therapeutic application into ER stress-involved diseases.

MSC-derived EVs contained various cytokines that regulate cell proliferation, migration and survival [52, 53]. Crucial components for the protective effects of A-EVs were not determined in the study. However, possible mediators can be suggested from previous analyses of our A-EVs contents [51, 54–56]. From antibody analysis of cytokine analysis, TIMPs and IGF-1 were detected with high levels at multiple times. TIMPs showed neuroprotective effects from various stress, including hypoxia-reoxygenation [57], neuroinflammation [58] and excitotoxicity [59] via regulation of calcium influx [59] or apoptosis signaling pathway [58, 60]. IGF1 also protects neurons from brain injury, stroke, and neuroinflammatory response [61]. To clarify these, detailed further mechanism studies are necessary.

Recent studies have demonstrated that mesenchymal stem cell (MSC)-derived EVs can promote neuronal survival, which can lead to neurogenesis, neuronal differentiation and neuronal regeneration and prevent neuronal apoptosis [36, 62]. Indeed, we found that A-EVs prevent neuronal apoptosis in CORT-induced cortical neurons. These data are consistent with previous studies that showed increased neuronal survival and prevention of neuronal apoptosis in hippocampal neuron cultures after A-EVs treatment [30, 63]. Furthermore, MSC-derived EVs alleviated the effects of stroke and brain injury by activating neurite remodeling, neurogenesis and angiogenesis in rodent models [64, 65]. EVs derived from dental pulp stem cells rescued 6-hydroxydopamine (6-OHDA)-induced apoptosis in human dopaminergic neurons [66]. MSC-derived EVs protected hippocampal neurons from oxidative stress and synaptic damage by Alzheimer’s disease-linked amyloid beta oligomers [67]. Adipose-derived MSCs EVs promote neurogenesis and neurite outgrowth in neurons via regulating various genes expression. Additionally, the adipose-derived MSCs rescue memory deficits in Alzheimer's model mice.[68]. Overall, the novel findings of the neuroprotective effects of MSC-derived EVs suggest an attractive therapeutic alternative for neurological and neurodegenerative diseases.

## Conclusions

We conclude from the present study that A-EVs ameliorates neuronal cell death induced by CORT in cultured cortical neurons. This study provides insight into the pathophysiological mechanisms of CORT and suggests that A-EVs could be useful in treating CORT-induced neuronal cell death.

## Data Availability

The supporting materials can be obtained upon request via email to the corresponding author.

## Abbreviations

A-EVs: Adipose stem cell-derived extracellular vesicles
MSC-EVs: Mesenchymal stem cell-derived extracellular vesicles
EVs: Extracellular vesicles
CORT: Corticosterone
ER stress: Endoplasmic reticulum stress
GRP78: Glucose-regulated protein 78
CHOP: C/EBP-homologous protein
ATF4: Activation transcription factor 4
PERK: Protein kinase RNA-like endoplasmic reticulum kinase
eIF2α: Eukaryotic translation initiation factor 2 alpha
ISRIB: Integrated stress response inhibitor, inhibitor of phosphorylated eIF2α
Bax: Bcl2-associated X
Bcl2: B-cell lymphoma 2
TIMP: Tissue inhibitors of matrix metalloproteinase
IFG-1: Insulin-like growth factor 1
TUNEL assay: Terminal deoxynucleotidyl transferase (TdT) dUTP Nick-End Labeling assay
DAPI: 4’,6-Diamidino-2-phenylindole
